# Genetics of Plasma Soluble Receptor for Advanced Glycation End-Products and Cardiovascular Outcomes in a Community-based Population: Results from the Atherosclerosis Risk in Communities Study

**DOI:** 10.1371/journal.pone.0128452

**Published:** 2015-06-17

**Authors:** Nisa M. Maruthur, Man Li, Marc K. Halushka, Brad C. Astor, James S. Pankow, Eric Boerwinkle, Josef Coresh, Elizabeth Selvin, Wen Hong Linda Kao

**Affiliations:** 1 Department of Medicine, Division of General Internal Medicine, The Johns Hopkins University School of Medicine, Baltimore, Maryland, United States of America; 2 The Welch Center for Prevention, Epidemiology, and Clinical Research, The Johns Hopkins University, Baltimore, Maryland, United States of America; 3 Department of Epidemiology, The Johns Hopkins University Bloomberg School of Public Health, Baltimore, Maryland, United States of America; 4 Department of Pathology, The Johns Hopkins University School of Medicine, Baltimore, Maryland, United States of America; 5 Department of Medicine, The University of Wisconsin School of Medicine and Public Health, Madison, Wisconsin, United States of America; 6 Department of Population Health Sciences, The University of Wisconsin School of Medicine and Public Health, Madison, Wisconsin, United States of America; 7 Division of Epidemiology and Community Health, University of Minnesota, Minneapolis, Minnesota, United States of America; 8 Division of Epidemiology, University of Texas at Houston School of Public Health, Houston, Texas, United States of America; University of Miami, UNITED STATES

## Abstract

Plasma soluble Receptor for Advanced Glycation End-products (sRAGE) is a strong marker of vascular outcomes although evidence on the direction of association is mixed. Compared to whites, blacks have lower levels of sRAGE. We hypothesized that genetic determinants of sRAGE would help clarify the causal role of sRAGE and the black-white difference in sRAGE levels. We conducted a genome-wide analysis of sRAGE in whites and blacks from the Atherosclerosis Risk in Communities Study. Median plasma sRAGE levels were lower in blacks than whites (728 vs. 1067 pg/ml; P<0.0001). The T (vs. C) allele of rs2070600, a missense variant in *AGER*, the gene encoding RAGE, was associated with approximately 50% lower sRAGE levels in both whites (N = 1,737; P = 7.26x10^-16^; minor allele frequency (MAF) = 0.04) and blacks (N = 581; P = 0.02; MAF = 0.01). In blacks, the T (vs. C) allele of rs2071288, intronic to *AGER*, was associated with 43% lower sRAGE levels (P = 2.22x10^-8^; MAF = 0.10) and was nearly absent in whites. These *AGER* SNPs explained 21.5% and 26% of the variation in sRAGE in blacks and whites, respectively, but did not explain the black-white difference in sRAGE. These SNPs were not significantly associated with incident death, coronary heart disease, diabetes, heart failure, or chronic kidney disease in whites (N = 8,130–9,017) or blacks (N = 2,293–2,871) (median follow up ~20 years). We identified strong genetic determinants of sRAGE that did not explain the large black-white difference in sRAGE levels or clearly influence risk of clinical outcomes, suggesting that sRAGE may not be a causal factor in development of these outcomes.

## Introduction

The soluble receptor for advanced glycation end-products (sRAGE) has emerged as a biomarker of cardiovascular disease [1–5] and all-cause mortality [4–7]. In prospective studies, blood levels of sRAGE have been associated with important cardiovascular outcomes, but the direction of associations has varied. Some studies have demonstrated an association between higher sRAGE levels and incident cardiovascular disease [1, 5], cardiovascular mortality [2], and all-cause mortality [2, 5], but others have reported a significant inverse association between sRAGE and cardiovascular disease [4], progression of carotid atherosclerosis by intima media thickness [3], and all-cause mortality [4, 7].

An additional striking finding from studies of sRAGE in humans is the substantial difference among racial and ethnic groups. In the Atherosclerosis Risk in Communities Study (ARIC), Dallas Heart Study, and the Northern Manhattan Study, non-Hispanic blacks [4, 8] and Hispanics [9] had significantly lower sRAGE levels than whites, and adjustment for multiple confounding variables did not change this racial difference in sRAGE levels [4, 9], leading to a hypothesis that genetic factors may explain at least a proportion of the observed racial difference. Given the strong associations of sRAGE with clinical outcomes, understanding this racial difference could have implications for racial disparities in cardiovascular disease.

To clarify mixed findings of large associations between sRAGE and outcomes in prior studies and to further understand the black-white difference in sRAGE levels, we used the Atherosclerosis Risk in Communities (ARIC) Study, a multi-ethnic, community-based cohort study to 1) conduct genome-wide association studies (GWAS) of sRAGE levels in whites and blacks separately; 2) evaluate if genetic variation underlies the black-white difference in sRAGE; and 3) conduct a Mendelian randomization study to evaluate the association between genetic determinants of sRAGE and mortality, coronary heart disease, congestive heart failure, diabetes, and chronic kidney disease. We hypothesized that we would identify genetic determinants of sRAGE, that genetic variation that would explain at least part of the black-white difference in sRAGE levels, and that associations between genetic determinants of sRAGE and vascular outcomes would inform the causal role of sRAGE in cardiovascular disease and related outcomes.

## Methods

### Study Population

The ARIC Study is a community-based prospective cohort of 15,792 individuals recruited from 4 US communities (Forsyth County, NC; Jackson, MS; suburban Minneapolis, MN; and Washington County, MD) who were between the ages of 45 and 64 years at enrollment in 1987 to 1989. After enrollment, there were three follow-up visits approximately every three years (1990–92, 1993–95, 1996–98). A fifth visit was completed from 2011 to 2013. Details of the ARIC cohort have been published elsewhere [10].

sRAGE was measured in a random sample of 2,024 participants at visit 2 and also measured in 1,019 participants (as part of an ancillary study consisting of persons with diabetes and a random sample of persons without diabetes) at visit 1. We excluded samples from visit 1 for participants who had sRAGE measured at both visit 1 and visit 2 (N = 134), participants without GWAS genotyping data (N = 392), without genotyping samples that passed quality control measures (N = 188), or who had missing diabetes status at visit 2 (N = 11), resulting in 581 black and 1,737 white participants for the GWAS of sRAGE levels.

For the analysis of *AGER* (Gene ID: 177; Entrez Gene) SNPs and clinical outcomes, we were not limited to the ARIC subsample in which sRAGE was available and of the entire cohort of black and white participants with follow-up from visit 1 (1987–1989) who consented to non-cardiovascular disease-related research (N = 15,703), we excluded participants without genotyping data (N = 2,778) and those with genotyping samples that did not pass quality control measures (N = 1,037). For the analysis of each incident clinical outcome (see below), we excluded those with disease at Visit 1 (e.g., participants with prevalent diabetes were excluded from the analysis of incident diabetes) and those without data on the variables of interest.

Figs A and B in S1 File provide details on the analytic sample for each of these analyses.

### GWAS methods

Single-nucleotide polymorphisms (SNPs) were genotyped on the Affymetrix 6.0 platform and were imputed to ≈ 37 million SNPs based on a cosmopolitan reference panel of haplotypes from 1000 Genomes Phase I [11]. Imputation was done using IMPUTE version 2 and the pre-phasing step was done by using SHAPEIT2 [12]. Extensive quality control was performed, and individuals were excluded based on the following: SNP missing rate higher than 5%, gender mismatch, high discordance with previous Taqman assay genotypes, genetic outlier status, and relatedness. Principal components analysis using a subset of the GWAS SNPs was used to estimate population substructure with the software EIGENSTRAT [13]; 10 factors were estimated, and one was associated with sRAGE in whites while none were associated with sRAGE in blacks. The imputation, quality control analyses, and principal components analysis were done separately by race; after exclusion of SNPs with MAF <5% and those with r^2^ <0.3 (imputation score), approximately 9.1 million and 6.5million SNPs were analyzed in ARIC blacks and whites, respectively.

Given that SNP identifiers change over time, for consistency, we have used rs numbers from dbSNP (GRCh37.p10) and have provided both the dbSNP (GRCh37.p10) and 1000G SNPID identifiers for each SNP in Table A in S1 File.

### Measurement of sRAGE

sRAGE was measured by ELISA (R&D Systems, Minneapolis, MN) in stored plasma samples [4]. Plasma sRAGE levels have been shown to remain fairly stable within individuals over three years in the ARIC Study [14]. For participants with sRAGE at visit 1 only, we predicted visit 2 levels based on linear regression of sRAGE at visit 1 on age.

### Measurement of covariates

For analyses of the ARIC sub-sample with sRAGE levels (N = 2,318), covariates included self-reported age, sex, race (black or white), and educational level (less than high school, high school or any college, more than four years post-high school); body mass index calculated as measured weight/height^2^ in kg/m^2^ [15]; prevalent diabetes mellitus (self-reported diabetes, use of diabetes medications, and/or fasting glucose ≥6.99 mmol/l among those with fasting samples); estimated glomerular filtration rate (eGFR) calculated from measured creatinine [16] and age, sex, and race using the Modification of Diet in Renal Disease Study Group method [17]; and prevalent coronary heart disease by self-report or diagnosis by ARIC procedures described below. Fasting glucose was measured as described previously [16]. Sex, race, and education were reported at Visit 1, and all other covariates were from Visit 2 in this sample.

For the analyses of associations between genetic determinants of sRAGE and clinical outcomes (death, incident coronary heart disease (CHD), incident diabetes, incident chronic kidney disease (CKD), and incident heart failure), self-reported age, sex, race, and education (as categorized above) ascertained at Visit 1 were included as covariates.

### Clinical Outcomes

Deaths and incident CHD, CKD, diabetes, and heart failure were ascertained from in-person clinic visits, active surveillance for hospitalizations and deaths, and annual telephone calls to all participants or their proxies. Incident CHD was adjudicated by an endpoints committee and defined as the first definite or probable myocardial infarction (MI), fatal CHD or MI by electrocardiogram. Incident heart failure was defined by death or hospitalization for heart failure. Incident diabetes was defined by first self-report of diabetes, fasting glucose ≥6.99 mmol/l, or use of diabetes medications. Incident CKD was defined by first occurrence of eGFR <60 ml/min per 1.73 m^2^.

### Analysis

We used a two-sided t test to compare means (if normally-distributed) or Wilcoxon test to compare distributions (if not normally-distributed) and a chi-squared test to compare proportions of characteristics between blacks and whites and assessing associations of variables with sRAGE levels.

We conducted genome-wide association analyses of natural log-transformed sRAGE using linear regression with an additive genetic model in the ARIC white cohort and in the ARIC black cohorts separately using SNPTEST version 2.4.1 [18]. We adjusted for age, gender, center, diabetes status, and principal components that were associated with sRAGE (one in whites and none in blacks). SNPs with minor allele frequency (MAF) < 5% were excluded from the initial analysis. Regional association plots showing linkage disequilibrium (LD), recombination rates and the location of nearby genes were generated for the top ranking SNPs for each race using a 400 kb window [19]. Genome-wide significance was defined as a P-value <5×10^−8^.

To follow up our GWAS findings, we conducted additional analyses of SNPs at the *AGER* gene and its flanking regions based on their correlation with the most significant GWAS SNPs in whites and blacks (r^2^>0.06 and MAF≥1%). We also conducted conditional analyses using the most significant SNP from the GWAS in blacks and whites separately. We focused on the *AGER* gene for these additional analyses because of the biologic plausibility of this gene being causal as it encodes RAGE.

We selected the most significant SNP from the above analyses to evaluate the multivariate association of race (blacks vs. whites) with sRAGE levels using linear regression and included covariates associated with sRAGE.

We assessed the association of the top sRAGE SNP and clinical outcomes using Cox proportional hazards models in whites and blacks separately; these analyses were conducted using the R survival package, [20, 21] and P <0.05 was considered statistically significant.

We estimated the percentage of variance explained by a SNP using *R* ^*2*^ = *b* ^*2*^ *var(SNP)/var(ln(sRAGE))* where *b* is the estimated effect of the SNP on ln(sRAGE), and var(SNP) = 2*MAF*(1-MAF) [22]. We estimated var(ln(sRAGE)) in each ancestry group separately.

We performed a post-hoc power calculation for blacks and whites separately using the non-centrality-parameter-based method for estimating power in Mendelian randomization studies described by Brion et al, 2013 [23, 24] and estimated power for the analyses of clinical outcomes assuming the following: type I error rate of 0.05; proportion of variance in ln(sRAGE) explained by SNP as reported in this study; event rates observed in this study; odds ratios for ln(sRAGE) and the clinical outcomes estimated using logistic regression in this study (Table B in S1 File). With the exception of CKD, we estimate >80% power for all outcomes in whites and for all outcomes besides heart failure in blacks (Table B in S1 File).

Participants in this study provided written informed consent, and approval from Institutional Review Boards from participating institutions was obtained. The authors report no disclosures.

## Results

Baseline age, sex, education, body mass index (BMI), prevalence of CHD, and fasting glucose differed between black and white participants in ARIC having sRAGE values (N = 2,329; Table 1). Median sRAGE was 339 pg/l lower in blacks compared to whites (P = 1.26 x 10^−68^). sRAGE levels were lower among men and were lower with increasing BMI (Table C in S1 File).

**Table 1:** Characteristics of black and white participants with sRAGE levels (N = 2,329).^a^ ^,^ ^b^ ^,^ ^c^ ^,^ ^d^

	Whites	Blacks
**N**	1737	581
**sRAGE, pg/ml**	1066 (837,1355)	728 (542, 963)
**Age, years**	57.64 (5.7)	55.45 (5.6)
**Male**	783 (45)	182 (31)
**Education**		
**≤11 years**	266 (15)	208 (36)
**High school graduate**	771 (44)	170 (29)
**Attended college**	699 (40)	202 (35)
**Diabetes**	100 (6)	101 (17)
**BMI, kg/m** ^**2**^	27.8 (5.1)	30.8 (6.7)
**Prevalent CHD**	84 (5)	15 (3)
**eGFR, ml/min/1.73m** ^**2**^	76.6 (14.7)	86.9 (20.0)
**Fasting glucose, mmol/l**	5.75 (0.75)	6.23 (1.43)

^a^ Continuous variables reported as means (SD) and categorical variables as n (%). Median (p25, p75) provided for sRAGE.

^b^ Education, n = 1739 for whites and 588 for blacks; prevalent CHD, n = 1707 for whites and n = 577 for blacks; eGFR, n = 568 for blacks; fasting glucose, n = 1726 for whites and n = 586 for blacks

^c^ P<0.05 for differences between race groups for all characteristics

^d^ Abbreviations: sRAGE, soluble receptor for advanced glycation end-products; BMI, body mass index; CHD, coronary heart disease; eGFR, estimated glomerular filtration rate

**Fig 1:**
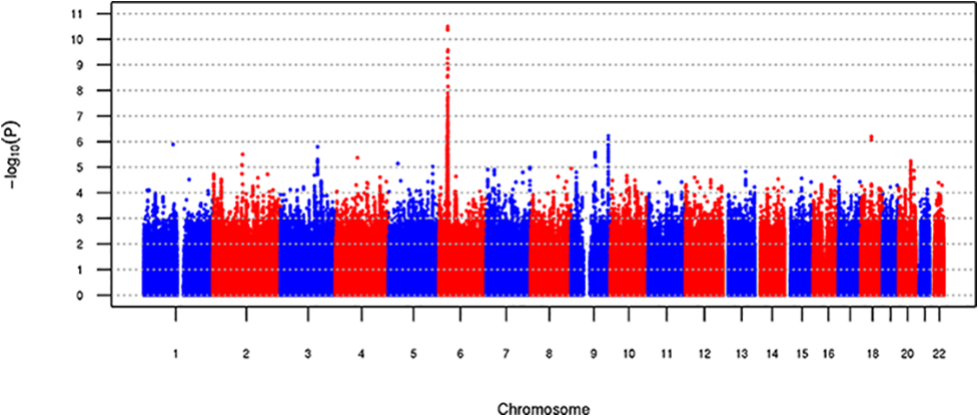
Manhattan plot for genome-wide association for ln(sRAGE) in whites (N = 1,737).

**Fig 2:**
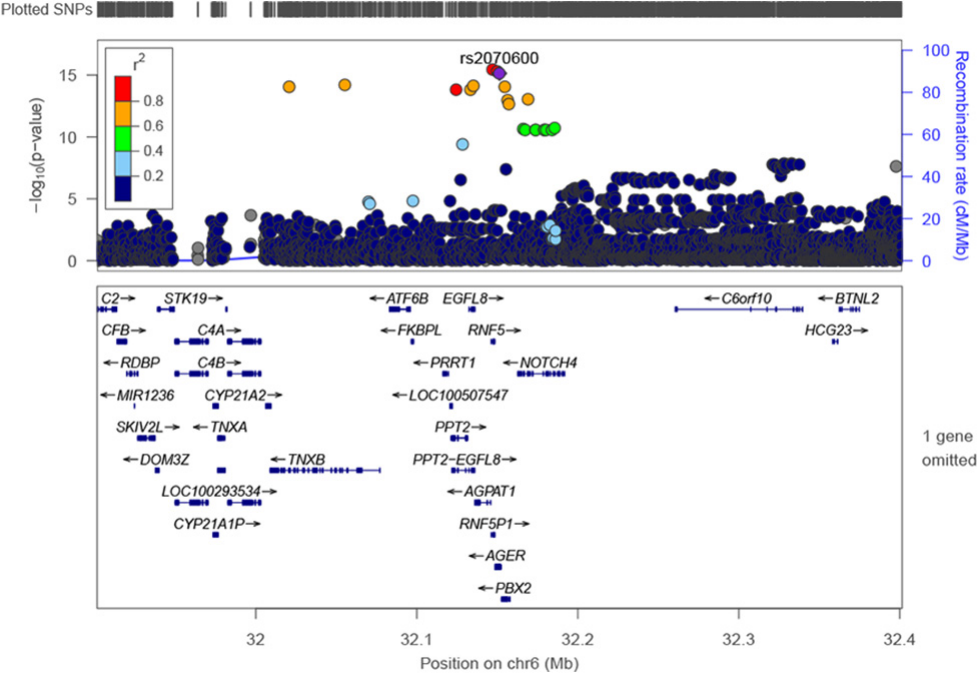
Regional association plot for genome-wide significant locus (*AGER*) in whites.

The genome-wide association study (GWAS) of natural log-transformed plasma sRAGE in white participants (N = 1,740) revealed a locus on chromosome 6 closest to the *NOTCH4* (Gene ID: 4855; Entrez Gene) gene (Fig 1) with rs2854050 having the most significant effect (P = 1.94 x 10^−11^, MAF = 0.06; Table 2). This area of chromosome 6 had several other genes nearby, including *AGER*, the gene encoding RAGE (Fig 2). Given the proximity of the *AGER* to *NOTCH4*, we conducted further analyses using SNPs with MAF ≥1% from the *AGER* gene (N = 29; Table A in S1 File) to determine whether the index SNPs in *NOTCH4* were tagging SNPs in *AGER*. Multiple *AGER* variants were associated with sRAGE levels in whites; a cluster of three highly-correlated SNPs were most significant (location range 32147044 to 32151443 (build 37); P values 2.7 x10^-16^ to 6.1 x 10^−16^; and MAF 0.042 to 0.044; Table D in S1 File). Of these three SNPs, rs2070600 (previously known as rs114177847), is a missense mutation (Gly→Ser; P = 6.08 x 10^−16^; Table 2). Our initial GWAS did not identify these SNPs because their MAFs were <5%. Rs2070600 is in high linkage disequilibrium (LD) with rs116807055 (*NOTCH4)* (r^2^ = 0.84 and D’ = 0.92). Conditional analyses of rs2070600 on *AGER* SNPs in whites did not reveal any other additional SNPs with genome-wide significance.

**Table 2:** Genome-wide significant loci and corresponding trans-ethnic results for sRAGE levels in whites and blacks.

SNP^a^	Gene	Chr:base pair position	Whites						Blacks				
			A1^b^/A2	A1 frequency	Β^c^	*P*	D’	r^2^ ^d^	A1 frequency	β	*P*	D’^e^	r^2^ ^e^
**rs2854050**	*NOTCH4*	6:32185605	A/G	0.06	-0.50	2.13e-11	NA	NA	0.03	-0.21	0.20301	1.0	0.003
**rs2071288**	*AGER*	6:32149260	T/C	0.005	-0.43	0.10	1.0	0.0	0.10	-0.56	2.22e-08	NA	NA
**rs2070600**	*AGER*	6:32151443	T/C	0.04	-0.67	7.26e-16	0.92	0.84	0.01	-0.63	0.017903	1.0	0.001

^a^From dbSNP build 37

^b^A1 is the minor allele in whites

^c^Mean change in ln(sRAGE) for Allele 1 vs. Allele 2

^d^With rs2854050 (index SNP from GWAS in whites)

^e^With rs2071288 (index SNP from GWAS in blacks)

**Fig 3:**
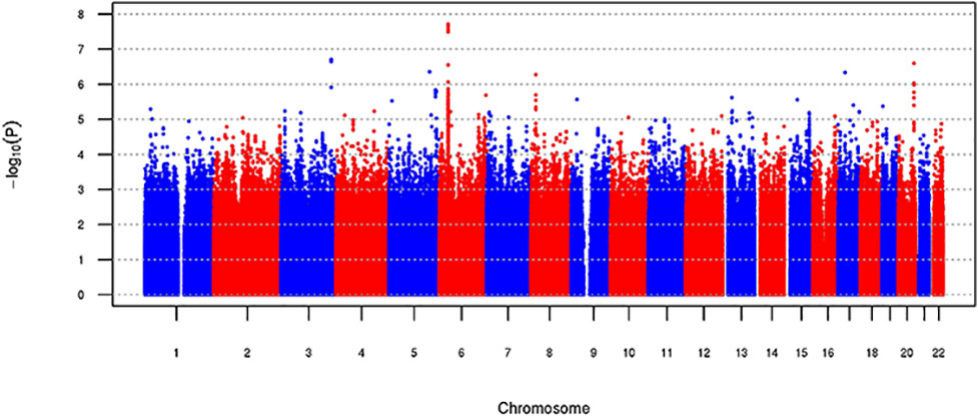
Manhattan plot for genome-wide association for ln(sRAGE) in blacks (N = 581).

**Fig 4:**
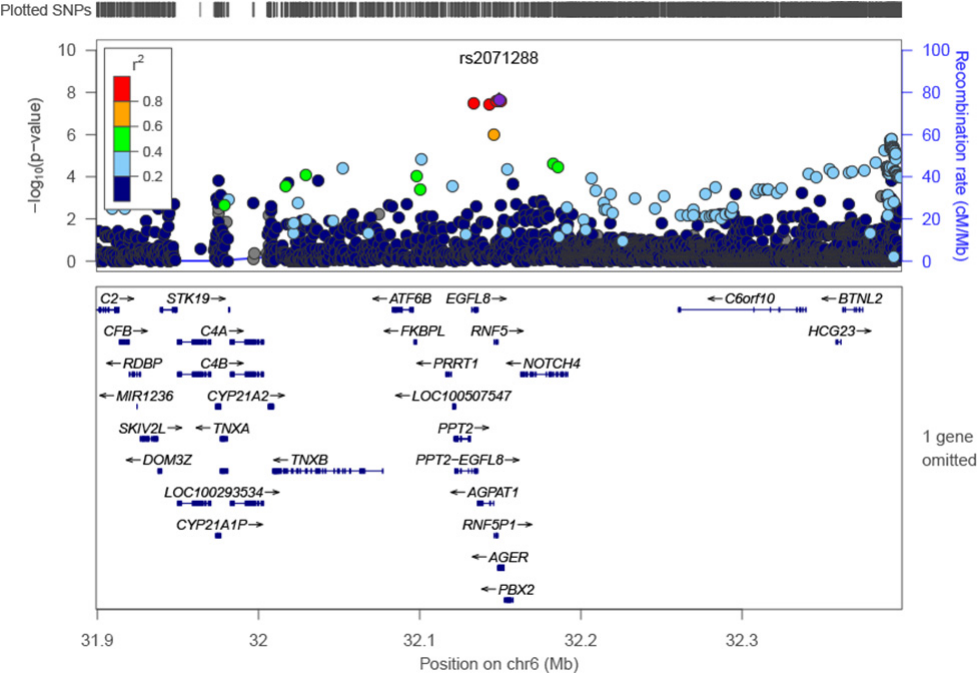
Regional association plot for genome-wide significant locus (*AGER*) in blacks.

In the GWAS conducted among black participants (N = 589; Figs 3 and 4), the most significant SNP was rs2071288 (P = 1.12 x 10^−8^; MAF = 0.10; Table 2), which is intronic to the *AGER* gene. The analysis of *AGER* SNPs extending the SNPs evaluated to those with MAF ≥1% (N = 29; Table A in S1 File) revealed a cluster of three highly-correlated SNPs on chromosome 6 which were most significant (location range 32147478 to 32150498 (build 37); P values 1.1x10^-8^ to 1.3x10^-8^; and MAFs 0.104 to 0.105; Table D in S1 File). Of these three SNPs, rs2071288 and rs114971929 are intronic (*AGER*) and the third one exonic and synonymous (*RNF5* (rs57409105)). Conditional analysis of rs2071288 on *AGER* SNPs in blacks did not reveal any other or additional SNPs reaching genome-wide significance. Rs2070600 was nominally associated with sRAGE levels in blacks (P = 0.018; Table 2) and remained nominally associated with sRAGE levels in blacks (P = 0.003) after adjustment for rs2071288.

The T allele of rs2070600 occurred in 4.4% of whites and 1.4% of blacks and was associated with 49 and 47% lower sRAGE in whites and blacks, respectively; rs2070600 explained 21.5% and 3.6% of the variation in ln(sRAGE) in whites and blacks, respectively. Rs2070600 was not in LD with the most significant GWAS SNP in blacks (Table 2). The T allele of the most significant SNP in blacks, rs2071288, intronic to *AGER*, was associated with a 43% reduction in sRAGE and explained 26% of the variability in ln(sRAGE) in blacks; this SNP was infrequent in whites (MAF = 0.005) and not significantly associated with ln(sRAGE) in whites. Rs2070600 and rs2071288 were not in LD with each other in either population (r^2^ of 0 and 0.001 in blacks and whites, respectively; Table 2). Accounting for these SNPs in regression models of the association between race (black vs. white) and lnsRAGE levels did not change the association between race and lnsRAGE. While adjustment for additional variables did decrease the beta coefficient for race by 0.1 ln units (18% absolute reduction), race remained the strongest predictor (by magnitude) of sRAGE levels in multivariate analyses (Table 3).

**Table 3:** Genetic variants and the black-white difference in ln(sRAGE).

	Model 1	Model 2	Model 3	Model 4
**Race (ref = white)**				
Beta for blacks	-0.58	-0.56	-0.56	-0.46
95% CI	-0.69 to -0.47	-0.67 to -0.45	-0.67 to -0.45	-0.56 to -0.35
P value	3.61E-24	8.40E-24	1.09E-23	8.50E-17
**rs2070600** ^**a**^ **(ref = C)**				
Beta for T allele		-0.26	-0.26	-0.27
95% CI		-0.32 to -0.20	-0.32 to -0.20	-0.33 to -0.21
P value		6.75E-16	5.48E-16	1.24E-18
**rs2071288** ^**a**^ **(ref = C)**				
Beta for T allele		-0.25	-0.25	-0.26
95% CI		-0.32 to -0.17	-0.32 to -0.17	-0.33 to -0.19
P value		3.01E-11	2.98E-11	6.40E-13

Model 1: age + race + gender + center

Model 2: Model 1 + rs2070600 + rs2071288

Model 3: Model 2 + education

Model 4: Model 3 + BMI, eGFR, fasting glucose, prevalent CHD

^a^Beta for difference in ln(sRAGE) for T vs. C allele

**Fig 5:**
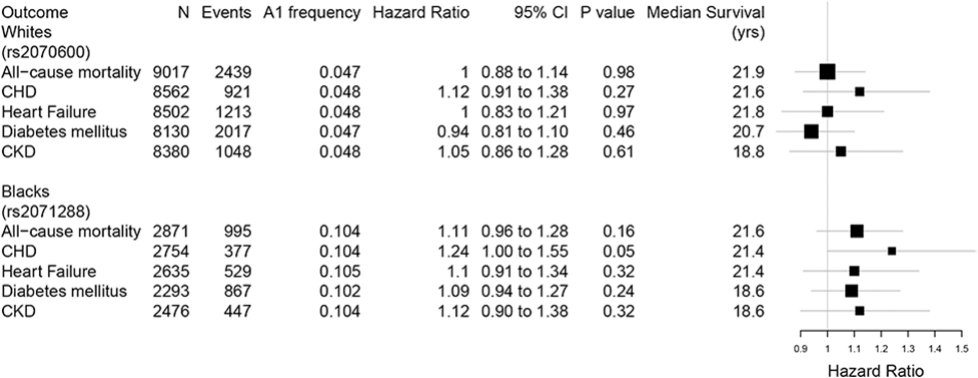
*AGER* SNPs and risk of death, incident coronary heart disease, heart failure, diabetes, and chronic kidney disease by race. Abbreviations: CHD, coronary heart disease; CKD, chronic kidney disease. Hazard ratio for T vs. C allele adjusted for age, sex, and center. Median follow up in years displayed.

Rs2070600 was not associated with all-cause death or incident coronary heart disease, heart failure, or diabetes in whites, and rs2071288 was not associated with these outcomes in blacks (Fig 5) although there was a marginal association between the T allele (sRAGE-lowering allele) and increased risk of CHD (HR, 1.24; 95%CI, 1.0 to 1.6; P = 0.054) (Fig 5). Of note, the study sample with sRAGE levels (N = 2,321) consisted of slightly more women, had a slightly higher BMI, lower prevalence of CHD, minimally lower eGFR, and lower fasting glucose compared to the sample included in these genetic association analyses without sRAGE levels (N = 9,590; Table E in S1 File).

## Discussion

Using a candidate gene approach to follow up our GWAS of plasma sRAGE levels, we identified rs2070600, a missense mutation in the *AGER* gene (Gly→Ser), as a variant influencing sRAGE levels in both blacks and whites. We also report a genetic determinant of sRAGE levels in blacks, rs2071288 which was associated with a 43% reduction in sRAGE levels and explained a substantial portion (26%) of the variability in lnsRAGE in this group. Rs2070600 was associated with ~50% lower levels of sRAGE in both whites and blacks and explained substantial (21.5%) and modest (3.6%) variation in this trait in these groups, respectively. Evaluation of rs2070600 and the most significant SNP in blacks, rs2071288, revealed that these genetic variants do not explain the observed black-white difference in sRAGE levels. Most importantly, rs2070600 and rs2071288 were not significantly associated with clinical outcomes, raising the suspicion that sRAGE may not the causal factor in prior studies implicating sRAGE in the development of [1, 2, 5] or protection from cardiovascular disease [4, 7].

Rs2070600 has been associated with sRAGE in prior candidate gene studies [25–28]. We used a non-biased genome-wide approach and confirmed rs2070600 as true genetic determinant of sRAGE levels. Moreover, with conditional analyses incorporating SNPs with MAF ≥1%, we were able to demonstrate that there is not another SNP which explains the effect of rs2070600. This evidence, in addition to biologic plausibility based on rs2070600 being a coding variant, supports that rs2070600 is a causal variant influencing sRAGE levels. The exact mechanism by which rs2070600 influences sRAGE levels is unknown and may include transcription, expression, or alternative splicing of *AGER*.

Interestingly, rs2071288, intronic to *AGER* and a major determinant of sRAGE in our study, was previously associated with plasma sRAGE levels and the diffusing capacity of carbon monoxide in a subgroup of white participants in a prior candidate gene study, but results from other ethnic groups were not reported [26]. The MAF among whites was higher (0.019) in that study compared to ours [26].

Our results demonstrating the lack of a significant effect of *AGER* variation on cardiovascular outcomes in whites are consistent with most prior case-control studies [29, 30] and extend these findings using a prospective cohort design which addresses confounding and selection bias more fully. While some case-control studies have identified associations between *AGER* SNPs and myocardial infarction and stroke [31] and prevalent coronary heart disease based on angiographic evidence of stenosis >50% [32], two recent meta-analyses including over 25 case-control studies (maximum N for single SNP = 7,111) [29] did not find a consistent association between *AGER* polymorphisms and coronary heart disease (odds ratios from meta-analyses ranged from 0.97 to 1.16 with *P* >0.05) [29, 30]. It is important to note, however, that rs2070600 has been associated with other diseases, including type 1 diabetes [33], chronic obstructive pulmonary disease [26], schizophrenia [34], and Alzheimer’s Disease [35].

The receptor for advanced glycation end products (RAGE) is a cellular receptor with multiple ligands, including advanced glycation end products (AGEs) and S100/calgranulins, and is expressed in a wide variety of cell types including endothelial cells, smooth muscle cells, phagocytes and neurons [36]. Interactions between RAGE and its ligands activate multiple signaling pathways which affect many important cell functions, ranging from the release of inflammatory cytokines to apoptosis depending on the tissue [36]. Excessive interactions between RAGE and its ligands may lead to cellular dysfunction [36]. sRAGE is a circulating, soluble form of RAGE which prevents binding of RAGE ligands to cellular RAGE thereby serving as a decoy for ligands and preventing these RAGE-ligand interactions [37] and possibly preventing cellular dysfunction. sRAGE is known to have at least two isoforms, a cleaved isoform caused by cleavage of the receptor from the cell membrane, and a spliced isoform (esRAGE) arising from alternative splicing of mRNA [38]. While it may be that one of these isoforms in particular or their regulation is relevant to pathophysiology (or “causal” in the sRAGE-RAGE axis), the standard assay method for sRAGE does not distinguish between these [38], and therefore we cannot assess the separate role of either isoform in incidence of the outcomes studied. Additionally, multiple isoforms of the membrane form of RAGE also exist and function differently, including in their affinity for different ligands [38].

Based on the results of our Mendelian randomization analysis, for the clinical outcomes in this study, we hypothesize that another causal factor is highly correlated with sRAGE in the complex pathway involving RAGE. The multi-ligand nature of RAGE and the multiple possible isoforms of RAGE and sRAGE [38], possibly resulting from particular disease states (e.g., the availability of advanced glycation end-products as ligands in diabetes), set the stage for complex interactions in the RAGE-sRAGE axis which likely cannot be marked by the current sRAGE measure.

Thus, our inability to determine a genetic basis to at least partly explain the black-white difference in sRAGE could result from the inability of sRAGE, as a biomarker, to represent the complex RAGE-ligand interaction. However, the lack of an identifiable genetic determinant of the racial difference in sRAGE in combination with a lack of an association between principal components and sRAGE in blacks suggests that environmental factors could be responsible for this difference. RAGE ligands are pro-inflammatory, and inflammation, as measured by C-reactive protein [39–41] and interleukin-6, is known to be higher among blacks vs. whites [41]. In our study, BMI and fasting glucose were higher in black vs. white participants; outside of known racial disparities in these factors and their downstream inflammatory diseases (e.g., diabetes and coronary heart disease), proximal environmental factors suggested to underlie the observed increased inflammation among blacks include lower socioeconomic status [39, 41], stress [42] and racial discrimination [43].

Of note, we did find that sRAGE levels were 14% lower among men compared to women and negligibly (1%) lower per unit increase in BMI; adjustment for these variables did not significantly impact the observed black-white difference in sRAGE levels.

The Mendelian randomization approach to determine if an exposure is causal relies on three major assumptions: 1) the genetic variant is associated with the exposure; 2) the genetic variant is associated with the outcome only through the exposure; and 3) the genetic variant is independent of confounders of the exposure-outcome association [44]. In our study, we have clearly shown an association between rs2070600 and sRAGE levels. While we cannot prove absolutely that rs2070600 would only affect the outcomes of interest through sRAGE, we can be confident that this would be the case given that rs2070600 is a SNP in the *AGER* gene which encodes sRAGE. Finally, we have adjusted our analyses for potential confounders. In light of the aforementioned assumptions, it has been suggested that Mendelian randomization analyses may actually be more useful for establishing a lack of causality, as we have in this study, rather than establishing causality [44].

A major strength of our study is that it is the most comprehensive evaluation of the genetics of sRAGE to date. We were able to identify a variant with genome-wide significance in whites for sRAGE and confirm our results in blacks. Our additional analyses establish that the coding *AGER* variant, rs2070600, is likely the most important genetic determinant of sRAGE levels in whites. We also identified an additional determinant of sRAGE levels in blacks, an intronic SNP (rs2071288) which was not correlated with rs2070600, and rs2071288 was associated with sRAGE in whites in a prior study[26] implying the likelihood of another independent genetic determinant of sRAGE levels in the *AGER* gene region. Finally, we were able to use this information to evaluate the causal role of sRAGE, as currently measured, in important outcomes–death, incident CHD, heart failure, diabetes mellitus, and CKD–using a Mendelian randomization design. Our post-hoc power calculations suggest that we had substantial power (power >80%) for these analyses of clinical outcomes in whites with the exception of the outcome of CKD (power <20%); as expected given the smaller sample size, we had lower power for some outcomes (CKD and heart failure) in blacks. Based on these power calculations and our findings, sRAGE does not appear to be a substantial and significant causal factor for death, heart failure, or diabetes in whites, and our results in blacks were similar. We did not have sufficient power to rule out a modest association between the sRAGE SNPs and CKD in blacks or whites; therefore, we cannot exclude the possibility of a causal role for sRAGE in CKD. Also, in blacks, the association between the sRAGE SNP (rs2071288) and incident CHD (HR 1.24, 95% CI, 1.00 to 1.55) nearly reached significance (P = 0.05), and this analysis was underpowered (power = 52%). For the sRAGE SNP in whites (rs2070600), we found a non-significant association in the same direction (HR 1.12, 95% CI, 0.91 to 1.38).

Our GWAS was limited to currently-identified variants meeting our quality control criteria, including the requirement for a SNP to occur at a frequency of at least five percent in the study population; we did do additional analyses to include SNPs with MAF ≥1%. However, this analysis would overlook rare variants, although it is unlikely that rare variants alone could explain the phenotypic difference in sRAGE between blacks and whites in this sample. We did select a single SNP from each racial group for our analyses of racial differences in sRAGE and clinical outcomes. Given the high correlation of the excluded SNPs with the selected SNPs, we are unable to exclude if they are the causal variants rather than the ones selected; we did select the SNPs used in the analysis based on statistical significance and proximity to the *AGER* locus, increasing biologic plausibility. Also, our evaluation of the genetic determinants of sRAGE does not include gene-environment or gene-gene interactions which could also explain variability in sRAGE and clinical outcomes.

## Conclusions

Through a GWAS, we have established the rs2070600 SNP, a coding variant in the *AGER* gene, as a likely causal determinant of sRAGE levels, but genetic variation in *AGER* does not appear to explain black-white difference in sRAGE. Also, strong genetic determinants of sRAGE levels were not associated with mortality, heart failure, or diabetes. We did not find evidence for robust associations between genetic determinants of sRAGE and either CKD or CHD, but we cannot rule out the possibility of modest positive effects given more limited power for these outcomes. These findings do not negate the importance of the RAGE-sRAGE axis but instead indicate that further study must identify the relevant causal factors for this axis given the strong, but mixed, epidemiologic associations observed between sRAGE and cardiovascular outcomes in prior studies [1, 2, 4, 5, 7]. Since sRAGE appears to be correlated with important clinical outcomes, the racial difference in sRAGE levels is likely relevant to the known racial disparities in cardiovascular disease, and given the lack of a clear genetic explanation, environmental factors identified in future studies could help address these disparities.

## Supporting Information

S1 File Supporting information. Selection of sample for sRAGE analyses (**Fig A**). Selection of sample for genetic association analyses (**Fig B**). *AGER* SNP identifiers (**Table A**). Power calculations for analyses of *AGER* SNPs and clinical outcomes (**Table B**). Bivariate associations with sRAGE levels (N = 2329) (**Table C**). Additional genome-wide significant loci and corresponding trans-ethnic results for sRAGE levels in whites and blacks (**Table D**). Characteristics of participants with sRAGE levels and participants included in analyses of clinical outcomes (**Table E**).
(DOCX)
