# Supplementary material for: Genetics of Plasma Soluble Receptor for Advanced Glycation End-Products and Cardiovascular Outcomes in a Community-based Population: Results from the Atherosclerosis Risk in Communities Study
Source: PLoS One. 2015 Jun 17;10(6):e0128452. doi: 10.1371/journal.pone.0128452 (PMC4471120; doi:10.1371/journal.pone.0128452)
Supplement: S1 File — Selection of sample for sRAGE analyses (Fig A). Selection of sample for genetic association analyses (Fig B). AGER SNP identifiers (Table A). Power calculations for analyses of AGER SNPs and clinical outcomes (Table B). Bivariate associations with sRAGE levels (N = 2329) (Table C). Additional genome-wide significant loci and corresponding trans-ethnic results for sRAGE levels in whites and blacks (Table D). Characteristics of participants with sRAGE levels and participants included in analyses of clinical outcomes (Table E). (DOCX) [file pone.0128452.s001.docx]

**Supporting Information**

**Table A**. ***AGER* SNP identifiers**

| **1000G SNPID** | **dbSNP (GRCh37.p10)** |
| --- | --- |
| rs146434120 | rs146434120 |
| chr6:32148909:D | NA |
| rs191750543 | rs191750543 |
| rs181811810 | rs181811810 |
| rs143357175 | rs143357175 |
| rs115535092 | rs2071288 |
| rs114564020 | rs114564020 |
| rs148720361 | rs3134941 |
| rs115168643 | rs2853807 |
| rs147062909 | rs55640627 |
| rs116515025 | rs9391855 |
| rs115963005 | rs3134940 |
| rs116334026 | rs204996 |
| rs115696666 | rs77170610 |
| rs186754929 | rs186754929 |
| chr6:32150272:I | NA |
| rs116217240 | rs184003 |
| rs114971929 | rs17846798 |
| rs189984228 | rs17846810 |
| rs182584636 | rs17846809 |
| rs142232164 | rs142232164 |
| rs1035798 | rs1035798 |
| rs146383902 | rs2269422 |
| rs114062306 | rs35795092 |
| rs114177847 | rs2070600 |
| rs116828224 | rs80096349 |
| rs115111668 | rs115111668 |
| rs115914022 | rs3131300 |
| rs115663512 | rs1800684 |

**Table B. Power calculations for analyses of *AGER* SNPs and clinical outcomes^[[1]](#endnote-1),^^[[2]](#endnote-2)^**

|  | **Type 1 error** | **N** | **Events** | **Proportion of variance in ln(sRAGE) explained by SNP** | **OR^[[3]](#endnote-3)^** | **Power^[[4]](#endnote-4)^** |
| --- | --- | --- | --- | --- | --- | --- |
| **Whites (rs2070600)** |  |  |  |  |  |  |
| All-cause death | 0.05 | 9017 | 2439 | 0.22 | 0.52 | 1 |
| CHD | 0.05 | 8562 | 921 | 0.22 | 0.79 | 0.83 |
| Heart Failure | 0.05 | 8502 | 1213 | 0.22 | 0.61 | 1 |
| Diabetes mellitus | 0.05 | 8130 | 2017 | 0.22 | 0.57 | 1 |
| CKD | 0.05 | 8380 | 1048 | 0.22 | 1.07 | 0.17 |
| **Blacks (rs2071288)** |  |  |  |  |  |  |
| All-cause death | 0.05 | 2871 | 995 | 0.26 | 0.52 | 1 |
| CHD | 0.05 | 2754 | 377 | 0.26 | 0.79 | 0.52 |
| Heart Failure | 0.05 | 2635 | 529 | 0.26 | 0.61 | 0.99 |
| Diabetes mellitus | 0.05 | 2293 | 867 | 0.26 | 0.57 | 1 |
| CKD | 0.05 | 2476 | 447 | 0.26 | 1.07 | 0.1 |

**Table C. Bivariate associations with sRAGE levels (N =2329)^[[5]](#endnote-5)^**

|  | Beta for ln(sRAGE) | 95% CI | P value |
| --- | --- | --- | --- |
| Age, years | -0.001 | (-0.004, 0.002) | 0.51 |
| Male | -0.15 | (-0.18, -0.11) | <0.0001 |
| White race | 0.48 | (0.37, 0.59) | <0.0001 |
| Education |  |  |  |
| ≤11 years | ref | - | - |
| High school or college | 0.08 | (-0.04, 0.05) | 0.73 |
| More than college | 0.03 | (-0.01, 0.08) | 0.17 |
| BMI | -0.01 | (-0.02, -0.01) | <0.0001 |
| Prevalent CHD | -0.05 | (-0.13, 0.03) | 0.26 |
| Prevalent diabetes |  |  |  |
| eGFR, mg/min/1.73m^2^ | -0.03 | (-0.004, -0.002) | <0.0001 |
| Fasting glucose, mmol/l | -0.01 | (-0.03, 0.01) | 0.18 |

**Table D**. **Additional genome-wide significant loci and corresponding trans-ethnic results for sRAGE levels in whites and blacks**

| **SNP^[[6]](#endnote-6)^** | **Gene** | **Chr:base pair position** | **Whites** |  |  |  |  |  | **Blacks** |  |  |  |  |
| --- | --- | --- | --- | --- | --- | --- | --- | --- | --- | --- | --- | --- | --- |
|  |  |  | A1^[[7]](#endnote-7)^/A2 | A1 frequency | β^[[8]](#endnote-8)^ | *P* | D’^[[9]](#endnote-9)^ | r^2^d | A1 frequency | β | *P* | D’^[[10]](#endnote-10)^ | r^2^e |
| **rs2070600** | *AGER* | 6:32151443 | T/C | 0.04 | -0.67 | 7.26E-16 | 0.92 | 0.84 | 0.01 | -0.63 | 1.79E-02 | 1.0 | 0.001 |
| [**rs41268928**](http://browser.1000genomes.org/Homo_sapiens/Variation/Summary?source=dbSNP;v=rs41268928) | *RNF5* | 6:32147157 | C/G | 0.04 | -0.69 | 3.46E-16 | 1 | 0.92 | 0.02 | -0.37 | 1.25E-01 | 1 | 0.001 |
| [**rs9391855**](http://browser.1000genomes.org/Homo_sapiens/Variation/Summary?source=dbSNP;v=rs9391855) | *AGER* | 6:32149801 | T/C | 0.04 | -0.67 | 5.06E-16 | 0.92 | 0.84 | 0.02 | -0.40 | 8.18E-02 | 1 | 0.001 |
| **rs2071288** | *AGER* | 6:32149260 | T/C | 0.005 | -0.43 | 1.00E-01 | 1.0 | 0.0 | 0.10 | -0.56 | 2.22E-08 | NA | NA |
| [**rs17846798**](http://browser.1000genomes.org/Homo_sapiens/Variation/Summary?source=dbSNP;v=rs17846798) | *AGER* | 6:32150498 | A/G | 0.005 | -0.43 | 1.01E-04 | 1 | 0 | 0.10 | -0.56 | 2.46E-08 | 1 | 1 |
| [**rs57409105**](http://browser.1000genomes.org/Homo_sapiens/Variation/Summary?source=dbSNP;v=rs57409105) | *RNF5* | 6:32147478 | T/G | 0.005 | -0.43 | 1.04E-01 | 1 | 0 | 0.10 | -0.56 | 2.60E-08 | 1 | 0.001 |

**Table E**. **Characteristics of participants with sRAGE levels and participants included in analyses of clinical outcomes^[[11]](#endnote-11),^^[[12]](#endnote-12)^**

|  | sRAGE sample | Genetic association sample^[[13]](#endnote-13)^ | P value |
| --- | --- | --- | --- |
| N | 2321 | 9590 |  |
| sRAGE, pg/ml | 983 (736.05,1279.81) | NA |  |
| Age, years | 57.06 (5.75) | 56.97 (5.70) | 0.48 |
| Male | 968 (42) | 4332 (45) | 0.002 |
| Education |  |  | 0.23 |
| ≤11 years | 475 (20) | 2056 (22) |  |
| High school graduate | 945 (41) | 3956 (41) |  |
| Attended college | 899 (39) | 3524 (37) |  |
| BMI | 28.5 (5.7) | 27.8 (5.3) | <0.001 |
| Prevalent CHD | 99 (4.3) | 545 (6.2) | 0.001 |
| eGFR | 79.3 (16.8) | 81.0 (17.8) | <0.001 |
| Fasting glucose | 5.89 (0.99) | 6.31 (2.28) | <0.001 |

**Figure A. Selection of sample for sRAGE analyses**

**Figure B. Selection of sample for genetic association analyses**


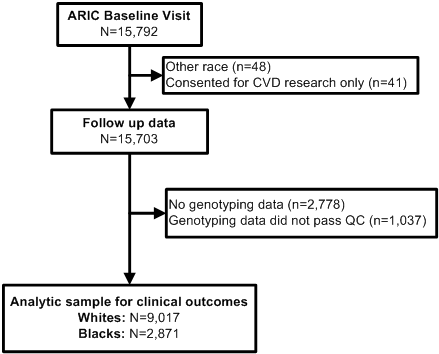


1. Type I error rate=0.05 for all calculations [↑](#endnote-ref-1)
2. Abbreviations: sRAGE, soluble receptor for advanced glycation end products; SNP, single nucleotide polymorphism; HR, hazard ratio [↑](#endnote-ref-2)
3. Odds ratio for ln(sRAGE) and outcome estimated using multivariate logistic regression with adjustment for age, sex, site (software: R). [↑](#endnote-ref-3)
4. Power calculated using Web tool (<http://glimmer.rstudio.com/kn3in/mRnd/>; Accessed 01/17/15) cited in Brion MJ, Shakhbazov K, Visscher PM: Calculating statistical power in Mendelian randomization studies. *Int J Epidemiol* 2013;42:1497-1501. [↑](#endnote-ref-4)
5. Abbreviations: sRAGE, soluble receptor for advanced glycation end-products; BMI, body mass index; CHD, coronary heart disease; eGFR, estimated glomerular filtration rate [↑](#endnote-ref-5)
6. From dbSNP build 37 [↑](#endnote-ref-6)
7. A1 is the minor allele in whites [↑](#endnote-ref-7)
8. Mean change in ln(sRAGE) for Allele 1 vs. Allele 2 [↑](#endnote-ref-8)
9. With rs2854050 (index SNP from GWAS in whites) [↑](#endnote-ref-9)
10. With rs2071288 (index SNP from GWAS in blacks) [↑](#endnote-ref-10)
11. Continuous variables reported as means (SD) and categorical variables as n (%). Median (p25, p75) provided for sRAGE. [↑](#endnote-ref-11)
12. Abbreviations: sRAGE, soluble receptor for advanced glycation end-products; BMI, body mass index; CHD, coronary heart disease; eGFR, estimated glomerular filtration rate [↑](#endnote-ref-12)
13. Subjects with sRAGE levels excluded [↑](#endnote-ref-13)
